# Supplementary figures and images for: New sources of Sym2A allele in the pea (Pisum sativum L.) carry the unique variant of candidate LysM-RLK gene LykX
Source: PeerJ. 2019 Nov 20;7:e8070. doi: 10.7717/peerj.8070 (PMC6874852; doi:10.7717/peerj.8070)

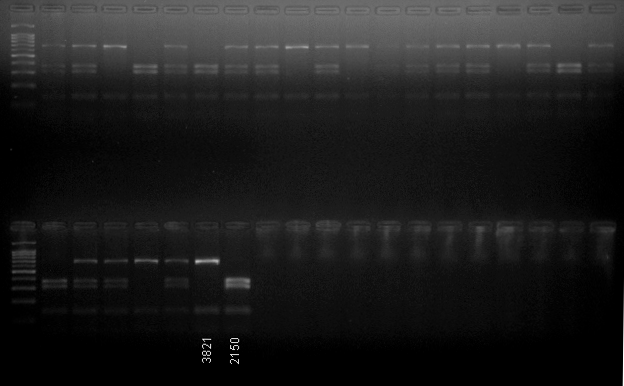

Supplement: Figure S1 — Part of the the F 2 (K-3821 x NGB2150) sample is shown. Parental lines are marked as 3821 and 2150. [file peerj-07-8070-s001.png]
