# Supplementary material for: New sources of Sym2A allele in the pea (Pisum sativum L.) carry the unique variant of candidate LysM-RLK gene LykX
Source: PeerJ. 2019 Nov 20;7:e8070. doi: 10.7717/peerj.8070 (PMC6874852; doi:10.7717/peerj.8070)
Supplement: Table S1 — Assessment of nodule formation has been performed qualitatively: 0 = “–”; <10 = “–/+”; 10-30 = “+/–”; 40–60 = “+”; 70–90 = “++”; >100 = “+++”. For all lines, the number of plants per pot has been 5. *: In (Zhukov et al., 2008), the line K-1887 (VIR1887) with the narrow symbiotic specificity from the ARRIAM collection has been mentioned. There has been probably a mistake either in ARRIAM or in VIR protocols, and these two lines are the same. Here, we keep the numeration of VIR collection. **: Line K-6566 has been lost during the work. [file peerj-07-8070-s002.docx]

| VIR accession number | Place of origin | Nodulation with *nodX^–^* strain (RCAM1026) | Nodulation with *nodX^+^* strain (A1) |
| --- | --- | --- | --- |
| K-73 | Pamir | + | + |
| K-108 | Turkey | ++ | ++ |
| K-188 | Tajikistan (Pamir) | ++ | ++ |
| K-236 | Afghanistan | ++ | + |
| K-864 | Kazakhstan | +/– | + |
| K-958 | Uzbekistan | + | + |
| K-1248 | Tajikistan | +/– | + |
| K-1250 | Tajikistan (Pamir) | ++ | ++ |
| K-1441 | Pamir | + | ++ |
| K-1837 | India | + | ++ |
| K-1852 | Palestine | + | + |
| K-1878* | Afghanistan | – | + |
| K-1974 | Afghanistan | + | + |
| K-2090 | India | +/– | + |
| K-2094 | Armenia | +/– | +/– |
| K-2095 | Armenia | ++ | + |
| K-2172 | Israel | + | + |
| K-2182 | Iran | –/+ | +/– |
| K-2198 | Turkey | + | + |
| K-2209 | Turkey | ++ | ++ |
| K-2223 | Turkey | ++ | ++ |
| K-2227 | Turkey | ++ | ++ |
| K-2240 | Turkey | + | +/– |
| K-2248 | Turkey | ++ | ++ |
| K-2258 | Turkey | + | + |
| K-2262 | Turkey | + | + |
| K-2309 | Georgia | + | + |
| K-2478 | Syria | + | + |
| K-2489 | Azerbaijan | +/– | + |
| K-2490 | Turkey | + | + |
| K-2496 | Turkey | + | ++ |
| K-2514 | Syria | +/– | + |
| K-2516 | Palestine | + | + |
| K-2521 | Israel | ++ | ++ |
| K-2524 | Lebanon | + | + |
| K-2595 | Israel | + | + |
| K-3208 | Turkey | + | + |
| K-3244 | Georgia | ++ | ++ |
| K-3264 | Turkey | ++ | + |
| K-3374 | Turkmenistan | – | + |
| K-3434 | India, Tibet | ++ | – |
| K-3567 | Yemen | +++ | + |
| K-3598 | Pamir | +++ | ++ |
| K-3607 | Pamir | + | ++ |
| K-3785 | Georgia | + | ++ |
| K-3821 | Tajikistan | – | ++ |
| K-3828 | Tajikistan | + | +++ |
| K-3829 | Tajikistan | + | ++ |
| K-3980 | Georgia | + | + |
| K-4379 | Kyrgyzstan | + | + |
| K-4396 | Kyrgyzstan | ++ | ++ |
| K-4650 | Armenia | + | + |
| K-4902 | Uzbekistan | – | + |
| K-5101 | Kyrgyzstan | + | ++ |
| K-5212 | India | + | + |
| K-5213 | India | + | +/– |
| K-5223 | India | ++ | +/– |
| K-5351 | India | +/– | + |
| K-5369 | Pakistan | +++ | ++ |
| K-5370 | Pakistan | ++ | + |
| K-5380 | Pakistan | + | + |
| K-5411 | Syria | ++ | + |
| K-5792 | Afghanistan | + | + |
| K-5804 | Afghanistan | + | + |
| K-5987 | India | + | + |
| K-6069 | Kazakhstan | ++ | ++ |
| K-6143 | Greece | + | ++ |
| K-6199 | Tajikistan | ++ | ++ |
| K-6201 | Tajikistan | + | ++ |
| K-6387 | Tajikistan | ++ | ++ |
| K-6452 | Turkey | + | + |
| K-6458 | Turkey | ++ | + |
| K-6528 | India | + | ++ |
| K-6559 | Afghanistan | – | ++ |
| K-6566** | Afghanistan | – | ++ |
| K-6650 | Pakistan | + | ++ |
| K-6651 | Pakistan | ++ | ++ |
| K-6653 | Pakistan | + | + |
| K-6654 | Pakistan | ++ | + |
| K-6657 | Pakistan | ++ | + |
| K-6872 | Kazakhstan | + | +++ |
| K-6982 | Kazakhstan | + | + |
| K-7005 | Syria | ++ | + |
| K-7006 | Syria | + | + |
| K-7008 | Syria | ++ | + |
| K-7034 | Nepal | + | + |
| K-7163 | Lebanon | + | + |
| K-7169 | Turkey | + | + |
| K-7173 | Turkey | ++ | + |
| K-7328 | Turkey | +++ | + |
| K-7382 | Georgia | + | ++ |
| K-7648 | Pakistan | + | + |
| K-7653 | Georgia | + | ++ |
| K-7656 | Pakistan | + | + |
| K-7657 | Pakistan | +++ | +++ |
| K-7786 | Russia, Dagestan | + | + |
| K-7814 | Kyrgyzstan | + | + |
| K-7861 | Kazakhstan | +/– | + |
| K-7868 | Georgia | ++ | + |
| K-7879 | Turkey | + | + |
| K-7892 | Turkey | + | + |
| K-7968 | Georgia | +++ | +++ |
| K-8025 | India | +++ | ++ |
| K-8026 | Yemen | +++ | ++ |
| K-8065 | Georgia | +++ | +++ |
| K-8067 | Georgia | + | + |
| K-8082 | Afghanistan | + | + |
| K-8095 | Pakistan | ++ | + |
| K-8258 | Georgia | +++ | +++ |
| K-8327 | Greece | + | + |
| K-8446 | Yemen | +/– | + |
| K-8536 | Greece | ++ | + |
| K-8546 | Afghanistan | ++ | ++ |
| K-8547 | Afghanistan | +++ | +++ |
| K-8563 | India | + | + |
| K-8645 | India | + | +/– |
| K-9066 | Syria | + | ++ |
| K-9135 | Syria | + | + |
| K-9205 | Greece | + | +/– |
| K-9282 | Tajikistan | ++ | + |
| K-9396 | Afghanistan | +/– | + |
| K-9398 | Iran | +/– | + |
