# Supplementary material for: New sources of Sym2A allele in the pea (Pisum sativum L.) carry the unique variant of candidate LysM-RLK gene LykX
Source: PeerJ. 2019 Nov 20;7:e8070. doi: 10.7717/peerj.8070 (PMC6874852; doi:10.7717/peerj.8070)
Supplement: Table S2 [file peerj-07-8070-s003.docx]

| **ARRIAM collection name** | **Synonyms** | **Place of origin** | **Symbiotic phenotype** | **Type** | **References** |
| --- | --- | --- | --- | --- | --- |
| 84 | K-6883,  VIR6883 | Uzbekistan | “Afghan” | Landrace | (Sulima et al., 2017) |
| cv. Iran | WBH 2149 (=NGB102149),  JI3001 | Iran | “Afghan” | Landrace | (Lie, 1971) |
| K-6047-2 | VIR6047-2 | Afghanistan | “Afghan” | Landrace | V.E. Tsyganov, A.Y. Borisov (personal communication) |
| NGB2150 | cv. Afghanistan,  WBH 2150 (=NGB102150), JI1357 | Afghanistan | “Afghan” | Landrace | (Lie, 1971; Holl, 1975; Kneen & LaRue, 1988; Tsyganov et al., 1999) |
| Caméor | JI3253 | France | “European” | Cultivar | (Dalmais et al., 2008) |
| Finale | JI2678 | The Netherlands | “European” | Cultivar | (Langille et al., 1981) |
| SGE | JI3023 | Russia | “European” | Laboratory line | (Kosterin & Rozov, 1993) |

**References**

Dalmais M, Schmidt J, Le Signor C, Moussy F, Burstin J, Savois V, Aubert G, Brunaud V, de Oliveira Y, Guichard C. 2008. UTILLdb, a Pisum sativum in silico forward and reverse genetics tool. *Genome biology* 9:R43.

Holl FB. 1975. Host plant control of the inheritance of dinitrogen fixation in the Pisum-Rhizobium symbiosis. *Euphytica* 24:767–770.

Kneen BE, LaRue TA. 1988. Induced symbiosis mutants of pea (Pisum sativum) and sweetclover (Melilotus alba annua). *Plant Science* 58:177–182.

Kosterin OE, Rozov SM. 1993. Mapping of the new mutation blb and the problem of integrity of linkage group I. *Pisum Genet* 25:27–31.

Langille JE, MacLeod JA, Smeltzer GG, Bubar JS. 1981. Finale field peas. *Canadian Journal of Plant Science* 61:1001–1002.
