# Supplementary material for: New sources of Sym2A allele in the pea (Pisum sativum L.) carry the unique variant of candidate LysM-RLK gene LykX
Source: PeerJ. 2019 Nov 20;7:e8070. doi: 10.7717/peerj.8070 (PMC6874852; doi:10.7717/peerj.8070)
Supplement: Table S3 — Concentrations are given for 1×working solution. Stock solutions are prepared separately for (1) MgSO 4 * 7H 2O, (2) K 2HPO 4, Ca 3(PO 4) 2, (3) NH 4NO 3, (4) NaFe-EDTA and (5) microelements, and then diluted to the required concentration. Stock solutions for (1), (2), (3), (4) are 100×. Stock solution for (5) is 1,000×. NH 4NO 3 is not to be added to the solution when analyzing the nodulation capability. [file peerj-07-8070-s004.docx]

| Component of solution | Concentration; mg/l | Component of solution | Concentration; mg/l |
| --- | --- | --- | --- |
| MgSO_4_ * 7H_2_O | 1000 | KI | 0.5 |
| K_2_HPO_4_ | 1000 | CuSO_4_ | 0.4 |
| Ca_3_(PO_4_)_2_ | 200 | Al_2_(SO_4_)_3_ * 18H_2_O | 0.3 |
| NH_4_NO_3_ | 600 | ZnSO_4_ * 7H_2_O | 0.2 |
| NaFe-EDTA | 10 | CoCl_2_ * 6H_2_O | 0.2 |
| microelements | | NaCl | 0,2 |
| H_3_BO_3_ | 5 | NiSO_4_ | 0.2 |
| (NH_4_)_2_MoO_4_ | 5 | MnSO_4_ | 0.2 |
| KBr | 0.6 | Li_2_SO_4_ | 0.2 |

**References**

Borisov AY, Rozov SM, Tsyganov VE, Morzhina E V., Lebsky VK, Tikhonovich IA. 1997. Sequential functioning of Sym-13 and Sym-31, two genes affecting symbiosome development in root nodules of pea (Pisum sativum L.). *Molecular and General Genetics* 254:592–598. DOI: 10.1007/s004380050456.
