# Supplementary material for: New sources of Sym2A allele in the pea (Pisum sativum L.) carry the unique variant of candidate LysM-RLK gene LykX
Source: PeerJ. 2019 Nov 20;7:e8070. doi: 10.7717/peerj.8070 (PMC6874852; doi:10.7717/peerj.8070)
Supplement: Table S4 — pH = 7.0–7.2 [file peerj-07-8070-s005.docx]

| Medium component | Concentration; g/l |
| --- | --- |
| K_2_HPO_4_ | 0.5 |
| MgSO_4_*7H_2_O | 0.2 |
| NaCl | 0.1 |
| CaCO_3_ | traces |
| Yeast extract | 0.4 |
| Mannitol | 10 |
| Agar-agar | 15 |
