# Supplementary material for: New sources of Sym2A allele in the pea (Pisum sativum L.) carry the unique variant of candidate LysM-RLK gene LykX
Source: PeerJ. 2019 Nov 20;7:e8070. doi: 10.7717/peerj.8070 (PMC6874852; doi:10.7717/peerj.8070)
Supplement: Table S5 — C1,2, control plants of cultivar Finale (“European” phenotype); M, mean; MC, mean for control plants. [file peerj-07-8070-s006.docx]

|  | **1** | **2** | **3** | **4** | **5** | **M** | **C1** | **C2** | **MC,**  **m±SD** |
| --- | --- | --- | --- | --- | --- | --- | --- | --- | --- |
| **NGB2150** | 0 | 0 | 0 | 0 | 0 | 0 | 43 | 40 | 41.5±2.1 |
| **K-6047-2** | 0 | 0 | 0 | 0 | 0 | 0 | 17 | 49 | 33±22.6 |
| **K-1878** | 0 | 0 | 0 | 0 | _ | 0 | 57 | _ | 57 |
| **K-4902** | 0 | 0 | 0 | 0 | 0 | 0 | 36 | 41 | 38.5±3.5 |
| **K-6559** | 0 | 0 | 0 | 0 | _ | 0 | 59 | _ | 59 |
| **K-3374** | 0 | 0 | 0 | 0 | 0 | 0 | 51 | _ | 51 |
| **K-3821** | 0 | 0 | 0 | 0 | 0 | 0 | 54 | _ | 54 |
| **F1 hybrids** | | | | | | | | | |
| **2150 x 6559** | 0 | 0 | 0 | 0 | _ | 0 | 40 | 39 | 39.5±0,7 |
| **6047-2 x 6559** | 0 | 0 | 0 | 0 | _ | 0 | 49 | 47 | 48±1.4 |
| **6047-2 x 1878** | 0 | 0 | 0 | 0 | _ | 0 | 56 | 16 | 36±28.3 |
| **4902 x 6047-2** | 0 | 0 | _ | _ | _ | 0 | 60 | 39 | 49.5±14.9 |
| **6559 x 1878** | 0 | 0 | 0 | 0 | 0 | 0 | 43 | 44 | 43.5±0.7 |
| **3821 x 2150** | 0 | 0 | 0 | _ | _ | 0 | 34 | 20 | 27±9.9 |
| **3374 x 2150** | 0 | 0 | 0 | 0 | _ | 0 | 16 | 62 | 39±32.5 |
