# Supplementary material for: New sources of Sym2A allele in the pea (Pisum sativum L.) carry the unique variant of candidate LysM-RLK gene LykX
Source: PeerJ. 2019 Nov 20;7:e8070. doi: 10.7717/peerj.8070 (PMC6874852; doi:10.7717/peerj.8070)
Supplement: Table S6 — The “allele” columns indicate the allelic state of LykX (A –“Afghan”, T –“Tajik”, H –heterozygote). The “nn” columns indicate the number of nodules formed by nodX − strain RCAM1026. One seed of cv. Finale was planted in each pot with four F 2 plants to ensure successful nodulation; number of nodules formed by cv. Finale is listed in “cv.Finale” column. The alleles frequency corresponds to 1:2:1 for monogenic segregation (chi-square = 0.025 (P = 0.99)). [file peerj-07-8070-s007.docx]

| **F_2_ hybrids (K-3821 x NGB2150)** | | | | | | | | | | | | **K-3821** | **NGB2150** | **cv. Finale** |
| --- | --- | --- | --- | --- | --- | --- | --- | --- | --- | --- | --- | --- | --- | --- |
| **№** | **allele** | **nn** | **№** | **allele** | **nn** | **№** | **allele** | **nn** | **№** | **allele** | **nn** | **nn** | **nn** | **nn** |
| **1** | A | 0 | **31** | H | 0 | **61** | H | 0 | **91** | Т | 0 | 0 | 0 | 37 |
| **2** | H | 0 | **32** | H | 0 | **62** | H | 1 | **92** | А | 0 | 0 | 0 | 73 |
| **3** | T | 0 | **33** | А | 0 | **63** | H | 0 | **93** | Т | 0 | 0 | 0 | 70 |
| **4** | H | 0 | **34** | H | 0 | **64** | Т | 0 | **94** | H | 0 | 0 | 0 | 55 |
| **5** | А | 0 | **35** | H | 0 | **65** | А | 2 | **95** | H | 0 |  | 0 | 59 |
| **6** | H | 0 | **36** | Т | 0 | **66** | А | 0 | **96** | А | 0 |  |  | 56 |
| **7** | Т | 0 | **37** | H | 0 | **67** | Т | 1 | **97** | Т | 0 |  |  | 85 |
| **8** | H | 0 | **38** | H | 0 | **68** | H | 0 | **98** | H | 0 |  |  | 46 |
| **9** | Т | 0 | **39** | H | 0 | **69** | H | 0 | **99** | А | 0 |  |  | 43 |
| **10** | Т | 0 | **40** | H | 0 | **70** | H | 0 | **100** | А | 0 |  |  | 112 |
| **11** | H | 0 | **41** | Т | 0 | **71** | А | 0 | **101** | Т | 0 |  |  | 74 |
| **12** | H | 0 | **42** | Т | 0 | **72** | Т | 0 | **102** | H | 0 |  |  | 38 |
| **13** | Т | 0 | **43** | Т | 0 | **73** | А | 0 | **103** | H | 0 |  |  | 82 |
| **14** | А | 0 | **44** | А | 0 | **74** | А | 0 | **104** | А | 0 |  |  | 60 |
| **15** | H | 0 | **45** | Т | 0 | **75** | Т | 0 | **105** | H | 0 |  |  | 38 |
| **16** | H | 0 | **46** | H | 0 | **76** | Т | 1 | **106** | H | 0 |  |  | 71 |
| **17** | H | 0 | **47** | А | 0 | **77** | H | 0 | **107** | А | 0 |  |  | 74 |
| **18** | А | 0 | **48** | А | 0 | **78** | А | 0 | **108** | Т | 1 |  |  | 83 |
| **19** | H | 0 | **49** | А | 0 | **79** | Т | 0 | **109** | H | 0 |  |  | 69 |
| **20** | H | 1 | **50** | Т | 0 | **80** | H | 0 | **110** | H | 0 |  |  | 52 |
| **21** | Т | 0 | **51** | H | 0 | **81** | Т | 0 | **111** | H | 0 |  |  | 87 |
| **22** | H | 0 | **52** | H | 0 | **82** | А | 0 | **112** | А | 0 |  |  | 93 |
| **23** | Т | 0 | **53** | H | 0 | **83** | H | 0 | **113** | H | 0 |  |  | 91 |
| **24** | H | 0 | **54** | H | 0 | **84** | H | 0 | **114** | H | 0 |  |  | 48 |
| **25** | H | 0 | **55** | Т | 0 | **85** | H | 0 | **115** | H | 0 |  |  | 26 |
| **26** | H | 0 | **56** | H | 0 | **86** | А | 0 | **116** | А | 0 |  |  | 70 |
| **27** | H | 0 | **57** | H | 0 | **87** | Т | 0 | **117** | H | 0 |  |  | 72 |
| **28** | Т | 0 | **58** | А | 0 | **88** | H | 0 | **118** | H | 0 |  |  | 63 |
| **29** | H | 0 | **59** | Т | 0 | **89** | Т | 0 | **119** | А | 0 |  |  | **m±SD** |
| **30** | А | 0 | **60** | H | 0 | **90** | А | 0 | **120** | - | - |  |  | 65.3 ± 20 |
